# Disruption of Ion-Trafficking System in the Cochlear Spiral Ligament Prior to Permanent Hearing Loss Induced by Exposure to Intense Noise: Possible Involvement of 4-Hydroxy-2-Nonenal as a Mediator of Oxidative Stress

**DOI:** 10.1371/journal.pone.0102133

**Published:** 2014-07-11

**Authors:** Taro Yamaguchi, Reiko Nagashima, Masanori Yoneyama, Tatsuo Shiba, Kiyokazu Ogita

**Affiliations:** Laboratory of Pharmacology, Faculty of Pharmaceutical Sciences, Setsunan University, Hirakata, Osaka, Japan; University of Louisville, United States of America

## Abstract

Noise-induced hearing loss is at least in part due to disruption of endocochlear potential, which is maintained by various K^+^ transport apparatuses including Na^+^, K^+^-ATPase and gap junction-mediated intercellular communication in the lateral wall structures. In this study, we examined the changes in the ion-trafficking-related proteins in the spiral ligament fibrocytes (SLFs) following *in vivo* acoustic overstimulation or *in vitro* exposure of cultured SLFs to 4-hydroxy-2-nonenal, which is a mediator of oxidative stress. Connexin (Cx)26 and Cx30 were ubiquitously expressed throughout the spiral ligament, whereas Na^+^, K^+^-ATPase α1 was predominantly detected in the stria vascularis and spiral prominence (type 2 SLFs). One-hour exposure of mice to 8 kHz octave band noise at a 110 dB sound pressure level produced an immediate and prolonged decrease in the Cx26 expression level and in Na^+^, K^+^-ATPase activity, as well as a delayed decrease in Cx30 expression in the SLFs. The noise-induced hearing loss and decrease in the Cx26 protein level and Na^+^, K^+^-ATPase activity were abolished by a systemic treatment with a free radical-scavenging agent, 4-hydroxy-2,2,6,6-tetramethylpiperidine 1-oxyl, or with a nitric oxide synthase inhibitor, N^ω^-nitro-L-arginine methyl ester hydrochloride. *In vitro* exposure of SLFs in primary culture to 4-hydroxy-2-nonenal produced a decrease in the protein levels of Cx26 and Na^+^, K^+^-ATPase α1, as well as Na^+^, K^+^-ATPase activity, and also resulted in dysfunction of the intercellular communication between the SLFs. Taken together, our data suggest that disruption of the ion-trafficking system in the cochlear SLFs is caused by the decrease in Cxs level and Na^+^, K^+^-ATPase activity, and at least in part involved in permanent hearing loss induced by intense noise. Oxidative stress-mediated products might contribute to the decrease in Cxs content and Na^+^, K^+^-ATPase activity in the cochlear lateral wall structures.

## Introduction

Mammalian cochlear spiral ligament (SL) fibrocytes (SLFs) of the mesenchymal non-sensory regions play important roles in the cochlear physiology of hearing. Their role includes the transport of K^+^ from the endolymph into the hair cells to generate the endocochlear potential (EP), which is essential for the transduction of sound by hair cells [1]–[3]. Once hair cells are activated by sound, the EP generated through the flow of K^+^ from the endolymph into the hair cells. It has been postulated that a K^+^-recycling pathway toward the stria vascularis (SV) via the SLFs in the cochlear lateral wall structures is critical for proper hearing, although the exact mechanism operating in this pathway has not been definitively determined [2]. Previous reports demonstrated that acoustic overstimulation produces alteration of the EP, as well as hearing loss [4], [5]. These findings suggest that acoustic injury is at least in part attributable to the abnormal EP induced by dysfunction of the SV and SL in the cochlear lateral wall structures. However, the mechanism underlying the acoustic overstimulation-induced dysfunction of the lateral wall structures is not fully understood.

Accumulating evidence indicates that gap junction (GJ)-mediated intercellular communication (GJ-IC) plays an important role in maintaining the unique ionic composition of the endolymph and intracellular ion content, both of which are crucial to cochlear functions. GJs are intercellular membrane channels that possess the unique feature of directly connecting the cytoplasm of neighboring cells. The GJ is formed by the juxtaposition of 2 hexameric structures (termed hemichannels or connexons) composed of connexins (Cxs) at the GJ plaques, where a large number of GJs cluster at the cell-cell contact points. Non-sensory cells in the cochlea are connected extensively by GJs that facilitate intercellular ionic and biochemical coupling for GJ-IC. The cochlear GJs are assembled with 2 subtypes of Cx family proteins, i.e., Cx26 and Cx30, which are widely distributed in the basal and intermediate cells of the SV, the supporting cells, the spiral limbus, and the spiral prominence [6], [7]. Evidence for involvement of Cxs in hearing ability comes from the finding that mutations in the Cx26-encoding gene (GJB2) cause at substantial portion (20–50%) of the cases of human non-syndromic hereditary deafness, which is one of the most common human birth defects. A large number of reports on human GJB2 mutations linked to prelingual deafness indicated loss-of-function mutations that effectively null the utility of Cx26 in the cochlea [8]. Disturbance of the GJ complex of Cx26 would be expected to disrupt the recycling of K^+^ from the synapses at the base of the hair cells through the supporting cells and SLFs in the cochlea. The disruption of K^+^ recycling is known to decrease sound-induced cochlear responses, resulting in sensorineural hearing loss [9]. In the present study, therefore, we evaluated whether Cx-related GJ-IC in the cochlear lateral wall structures is involved in the mechanism underlying noise-induced hearing loss.

Na^+^, K^+^-ATPase is a well-known enzyme that participates in the active transport of Na^+^ and K^+^, and plays important roles in maintaining cochlear function of the inner ear [10]–[12]. Cytochemical studies showed that strong activity of Na^+^, K^+^-ATPase is detectable in the SV and spiral prominence of the cochlear lateral wall structures, with the highest level of activity in the marginal cells of the SV [10], [13], [14]. However, little expression of Na^+^, K^+^-ATPase is found in the organ of Corti.

To the best of our knowledge, there have been very few reports regarding the functional role of Cxs in acquired sensorineural hearing loss. Although previous studies showed changes in the EP and K^+^ concentration in the endolymph after exposure to noise [4], [15], there has been no direct evidence for the involvement of a decrease in cochlear Na^+^, K^+^-ATPase activity in noise-induced hearing loss. In the present study, we investigated changes in Cx26 and Cx30 expression levels as well as Na^+^, K^+^-ATPase activity in the cochlear lateral wall structures following exposure to noise *in vivo*. Based on our previous study showing that exposure to noise *in vivo* produces 4-hydroxy-2-nonenal (4-HNE) in the lateral wall structures of the cochlea [16], we further investigated the *in vitro* effect of 4-HNE on the expression level of Cxs and on Na^+^, K^+^-ATPase activity in primary cultures of the SLFs.

## Materials and Methods

### Materials

Carbenoxolone, 4-hydroxy-2,2,6,6-tetramethylpiperidine-N-oxyl (tempol), and N^ω^-nitro-L-arginine methyl ester (L-NAME) were purchased from Sigma-Aldrich Co. (St. Louis, MO, U.S.A.). 4-HNE was supplied from Cayman Chemical (Ann Arbor, MI. USA). Mouse monoclonal antibodies against Cx26 and Na^+^, K^+^-ATPase α1 were obtained from Zymed Laboratories, Inc. (South San Francisco, CA, USA) and Santa Cruz Biotechnology, Inc. (Santa Cruz, CA, USA), respectively. Rabbit polyclonal antibodies against Cx30, S100β, and glyceraldehyde-3-phosphate dehydrogenase (GAPDH) were obtained from Zymed Laboratories, Inc. (South San Francisco, CA, USA), FabGennix Inc. (Frisco, TX, USA), and Santa Cruz Biotechnology, Inc. (Santa Cruz, CA, USA), respectively. Alexa-Fluor 598-conjugated anti-rabbit IgG (H+L) antibody, Alexa-Fluor 488-conjugated anti-mouse IgG (H+L) antibody, Calcein-AM, and 1,1-dioctadecyl-3,3,3′,3′-tetramethylindocarbocyanine perchloric acid (DiI) were purchased from Life Technology Co. (Carlsbad, CA, USA). Diaminobenzidine/hydrogen peroxide solution (Histofine) came from Nitirei Co. (Tokyo, Japan). Streptavidin-biotin complex peroxidase kit, poly-L-lysine hydrobromide, and trypsin solution were from Nacalai Tesque, Inc. (Kyoto, Japan). Polyvinylidene fluoride membranes (Immobilon-P) were obtained from Millipore (Bedford, MA, USA). Western Lightning Chemoluminescence Reagent Plus was purchased from PerkinElm1er Life Science Products, Inc. (Boston, MA, U.S.A.). All other chemicals used were of the highest purity commercially available.

### Animal treatment

The protocol used here met the guidelines of the Japanese Society for Pharmacology and was approved by the Committee for Ethical Use of Experimental Animals at Setsunan University. All efforts were made to minimize animal suffering, to reduce the number of animals used, and to utilize alternatives to *in vivo* techniques. Adult male Std-ddY mice weighing 26–28 g, which we routinely use for neuroscience studies, were housed in metallic breeding cages in a room with a light-dark cycle of 12 h–12 h and a humidity of 55% at 23°C and given free access to food and water. To remove animals with natural auditory impairment, we measured their auditory brainstem response (ABR) before use and selected those animals with normal acoustic sense in the present study.

### Intense noise exposure

The mice were anesthetized with chloral hydrate (500 mg/kg, i.p.) and exposed to 110-dB sound-pressure level (SPL) of octave-band noise, centered at 8 kHz, for 1 h within a sound chamber [17]. Each animal was placed in a cage. The sound chamber was fitted with a speaker (300HT; FOSTEX, Tokyo, Japan) driven by a noise generator (SF-06; RION, Tokyo, Japan) and power amplifier (DAD-M100proHT; FLYING MOLE, Shizuoka, Japan). To ensure uniformity of the stimulus, we calibrated and measured the sound levels with a sound- level meter (NL-26; RION, Tokyo, Japan), which was positioned at the level of the animal's head. As a control, naïve animals were placed in the same cage without noise.

### ABR recording

For ABR measurements, stainless steel-needle electrodes were placed at the vertex and ventro-lateral to the left and right ears. Electroencephalogram recording was performed with an extracellular amplifier Digital Bioamp system (BAL-1; Tucker-Davis Technologies, FL, USA), and waveform storing and stimulus control were performed by using Scope software of the Power Lab system (Power Lab 2/20; AD Instruments, Castle Hill, Australia). Sound stimuli were produced by a coupler-type speaker (ES1spc; Bioresearch Center, Nagoya, Japan) inserted into the external auditory canal of the mouse. Tone-burst stimuli, 0.1 ms rise/fall time (cosine gate) and 1-ms flat segment, were generated by using a Real-Time Processor (RP2.1; Tucker-Davis Technologies, FL, USA); and the amplitudes were specified by use of a Programmable Attenuator (PA5; Tucker-Davis Technologies, FL, USA). Sound levels were calibrated with a sound-level meter (TYPE 6224; ACO CO., LTD.). ABR waveforms were recorded for 12.8 ms at a sampling rate of 40,000 Hz by using 50–5000 Hz bandpass-filter settings. Waveforms from 500 stimuli were averaged. For recording, animals were anesthetized (500 mg/kg chloral hydrate, i.p.). The thresholds of ABR were determined before noise exposure and immediately (0) and on day 7 afterward at 12 kHz, by using a 5-dB SPL minimum step size down from the maximum amplitude. The hearing threshold was defined as the lowest stimulus intensity that produced a reliable wave I of the ABR. Because the constraining test tones at 4, 12, and 20 kHz were set to SPLs of less than 90 dB, the respective thresholds were recorded as 100 dB for the calculation of the threshold shift value when there was no response due to profound hearing impairment.

### Histological assessment

The mice were anesthetized deeply with chloral hydrate (500 mg/kg, i.p.) and intracardially perfused with saline and subsequently with 4% (wt/vol) paraformaldehyde in 0.1 M sodium phosphate buffer (pH 7.4). Their cochleae were removed quickly. The round and oval windows and the apex of the cochlea were opened and then perfused with Bouin's solution (picric acid: 37% (vol/vol) formaldehyde: acetic acid  = 15∶5∶1). The tissues were subsequently kept at room temperature overnight. The post-fixed cochlea was embedded in paraffin, and then sections at 5 µm thickness were prepared by using a microtome. The sections were deparaffinized with xylene and then rehydrated by passage through ethanol at graded concentrations of 50 to 100% (vol/vol) and then immersion in water. Immunoreactivity was determined by the avidin-biotin-peroxidase method. For the immunostaining of Cx26 and Cx30, the sections were washed with Tris-buffered saline (pH 7.5) containing 0.03% (wt/vol) Tween 20 (TBST) and then incubated with 0.03% (vol/vol) H_2_O_2_ in methanol for 5 min. After having been blocked with 10% (vol/vol) normal goat serum in TBST for 1 h at room temperature, serial sections were incubated with mouse monoclonal antibody against Cx26 (1∶200) or rabbit polyclonal antibody against Cx30 (1∶200) at 4°C overnight, followed by biotinylated anti-mouse IgG antibody or biotinylated anti-rabbit IgG antibody for 30 min at room temperature, and then with ABC solution for 1 h at room temperature. The peroxidase reaction was visualized by use of diaminobenzidine/hydrogen peroxide solution.

For double labeling with antibodies against Cx26 and Cx30 or S100β, sections covered with 10 mM sodium citrate buffer (pH 7.0) were first microwaved in a microwave oven for 10 min. After having been blocked with 10% (vol/vol) normal goat serum in TBST for 1 h at room temperature, the sections were incubated with primary antibodies against Cx26 and Cx30 or S100β at 4°C overnight. After having been washed with TBST, the sections were then reacted for 2 h at room temperature with secondary antibodies (Alexa Fluor 488-conjugated anti-mouse IgG for Cx26 and Alexa Fluor 598-conjugated anti-rabbit IgG for Cx30 or S100β). For double labeling with antibodies against Na^+^, K^+^-ATPase α1 and S100β, the sections were first microwaved as above and then blocked with 10% (vol/vol) normal goat serum in TBST for 1 h at room temperature, after which they were if the sections were first incubated with one primary antibody and then incubated with the other one, then “sequentially” would apply. But I assume that you would have used a mixture of both primary antibodies as the first step incubated with primary antibodies at 4°C overnight. After having been washed with TBST, the sections were next reacted for 2 h at room temperature with secondary antibodies (Alexa Fluor 488-conjugated anti-mouse IgG for Na^+^, K^+^-ATPase α1 and Alexa Fluor 598-conjugated anti-rabbit IgG for S100β). Finally, the sections were incubated with Hoechst 33342 (5 mg/mL) for 20 min at room temperature; and positive cells were determined by viewing with an Olympus U-LH100HG fluorescence microscope.

### Semi-quantitative RT-PCR and real-time PCR

For RT-PCR assays, total RNA was isolated from cortical neurons with Trizol by following the manufacturer's instructions (Invitrogen Co., California, USA). One microgram of total RNA was reverse transcribed to prepare cDNA by using Oligo(dT)_15_ primer in accordance with Ready-To-Go You-Prime First-Stranded Beads (GE Healthcare UK Ltd, Buckinghamshire, England). Aliquots of cDNA were then amplified with a 0.4 µM concentration of each primer set: 5′-AAATGTCTGCTATGACAAGTCCTTC-3′(forward) and 5′-CTTTGAGCTCCTCTTCTTTCTTGTT-3′(reverse) for the Cx43-encoding gene (GJA1); 5′-CCGTCTTCATGTACGTCTTTTACAT-3′ (forward) and 5′-ATACCTAACGAACAAATAGCACAGC-3′(reverse) for the Cx26-encoding gene (GJB2); 5′-AGTTTATACGTGGGGAGAAGAGAAA-3′ (forward) and 5′-TGGTACCCATTGTAGAGGAAGTAGA-3′(reverse) for the Cx30-encoding gene (GJB6); and 5′-GCCAAGTATGATGACATCAAGAAG-3′ (forward) and 5′-TCCAGGGGTTTCTTACTCCTTGGA-3′(reverse) for GAPDH in 25 µL of reaction mixture containing 0.2 mM concentration of each dNTP, 0.625 units Taq DNA polymerase, 10 mM Tris–HCl (pH 8.3), 50 mM KCl, 1.5 mM MgCl_2_, and 0.001% gelatin. Reactions were carried out for a total of 25–30 cycles with the use of a Thermal cycler, and then the PCR products were analyzed by performing1% agarose gel electrophoresis. For real-time PCR assays, an aliquot of cDNA was amplified by using a 0.2 µM concentration of each primer set for GJA1, GJB2, GJB6, and GAPDH genes in 12.5 µL of STBR Premix Ex Taq (Takara Bio Inc, Shiga, Japan).

### Immunoblot analysis

Cochlear SL structures were quickly removed and immersed in ice-cold homogenizing buffer consisting of 10 mM Tris-HCl buffer (pH 7.5), 0.32 M sucrose, 1 mM EDTA, 1 mM EGTA, 5 mM dithiothreitol, phosphatase inhibitors (10 mM sodium β-glycerophosphate and 1 mM sodium orthovanadate), and 1 µg/mL each of protease inhibitors [(p-amidinophenyl)methanesulfonyl fluoride, benzamidine, leupeptin, and antipain], homogenized in 30 µL of the homogenizing buffer, and then immediately boiled for 10 min in a solution comprising 2% (wt/vol) SDS, 5% (vol/vol) 2-mercaptoethanol, 10% (vol/vol) glycerol, and 0.01% (wt/vol) bromophenol blue. The samples were stored at −80°C until used for immunoblot analysis.

The immunoblot analysis was carried out as described previously [18]. Briefly, an aliquot (10 µg) of sample was loaded onto a 10% (wt/vol) polyacrylamide gel for detection of Cx26, Cx30, and GAPDH. The proteins were transferred to a polyvinylidene fluoride membrane and blocked with 5% (wt/vol) skim milk dissolved in washing buffer [Tris-buffered saline containing 0.05% (wt/vol) Tween 20]. The membranes were incubated with the desired primary antibody for 2 h at room temperature, then washed with the above washing buffer for 3 cycles of 5 min each, and subsequently incubated with horseradish peroxidase-conjugated antibodies against mouse IgG and rabbit IgG for 1 h at room temperature. Proteins reactive with the antibody were detected with the aid of Western Lightning Chemoluminescence Reagent Plus, which procedure was followed by exposure to X-ray films.

### Na^+^, K^+^-ATPase activity

Na^+^, K^+^-ATPase activity was determined by colormetrically measuring the amount of inorganic phosphate released from the substrate ATP [14]. Cochlear lateral wall structures were quickly removed and homogenized in ice-cold HEPES buffer (pH 7.5). An aliquot (6 µg protein) of the homogenate was incubated 10 min at 37°C in reaction buffer consisting of 40 mM Tris-HCl buffer (pH 7.5), 150 mM KCl, and 1.2 M NaCl in the absence or presence of 100 µM ouabain and then further incubated 30 min at 37°C in the reaction buffer containing 10 mM ATP and 30 mM MgSO_4_. The reaction was terminated by the addition of 12% perchloric acid and 0.84% ammonium molybdate to measure the released inorganic phosphate. The specific activity was calculated from the values obtained in the absence and presence of ouabain and expressed as µmol·min^−1^·mg protein^−1^.

### Primary cultures of SLFs

Mouse cochleae were removed from 5 animals under sterile conditions, and transferred to ice-cold Dulbecco's phosphate-buffered saline containing 100 U/mL penicillin, 0.1 mg/mL streptomycin, and 33 mM glucose. Following opening of the cochlear bone, the SL tissues were dissected with fine forceps. The SL tissues were cut into small longitudinal segments and then placed into type 1 collagen-coated Petri dishes (35 mm) containing 0.3 mL of culture medium consisting of DMEM supplemented with 10% fetal bovine serum (FBS), 100 U/mL penicillin, and 0.1 mg/mL streptomycin. At 3 and 4 days *in vitro* (DIV), 0.7 mL and 1 mL of fresh culture medium, respectively, was added to each Petri dish. The tissue segments were further cultured for 6 days with a change of the culture medium every 3 days. During the culture period, the cells migrated from the tissue segments and reached confluence at 10 DIV. The cells were then rinsed with phosphate-buffered saline and incubated with 0.25% trypsin solution for 5 min at 37°C to induce cell detachment. Culture medium was then added and gently triturated to suspend the cells. After centrifugation at 900×*g* for 5 min, the cells were washed twice with culture medium by gentle trituration and centrifugation. The cells were seeded at a density of 1,000 cells/0.5 mL in 4-well dishes (Nunc, Denmark) that had been previously coated with poly-L-lysine hydrobromide and then cultured for the desired periods with a change of culture medium every 3 days. Usually at 12 DIV, the cells were used for experiments. The cultures were always maintained at 37°C in 95% (vol/vol) air −5% (vol/vol) CO_2_.

### Fluorescent dye-transfer assay

**Figure 1:**
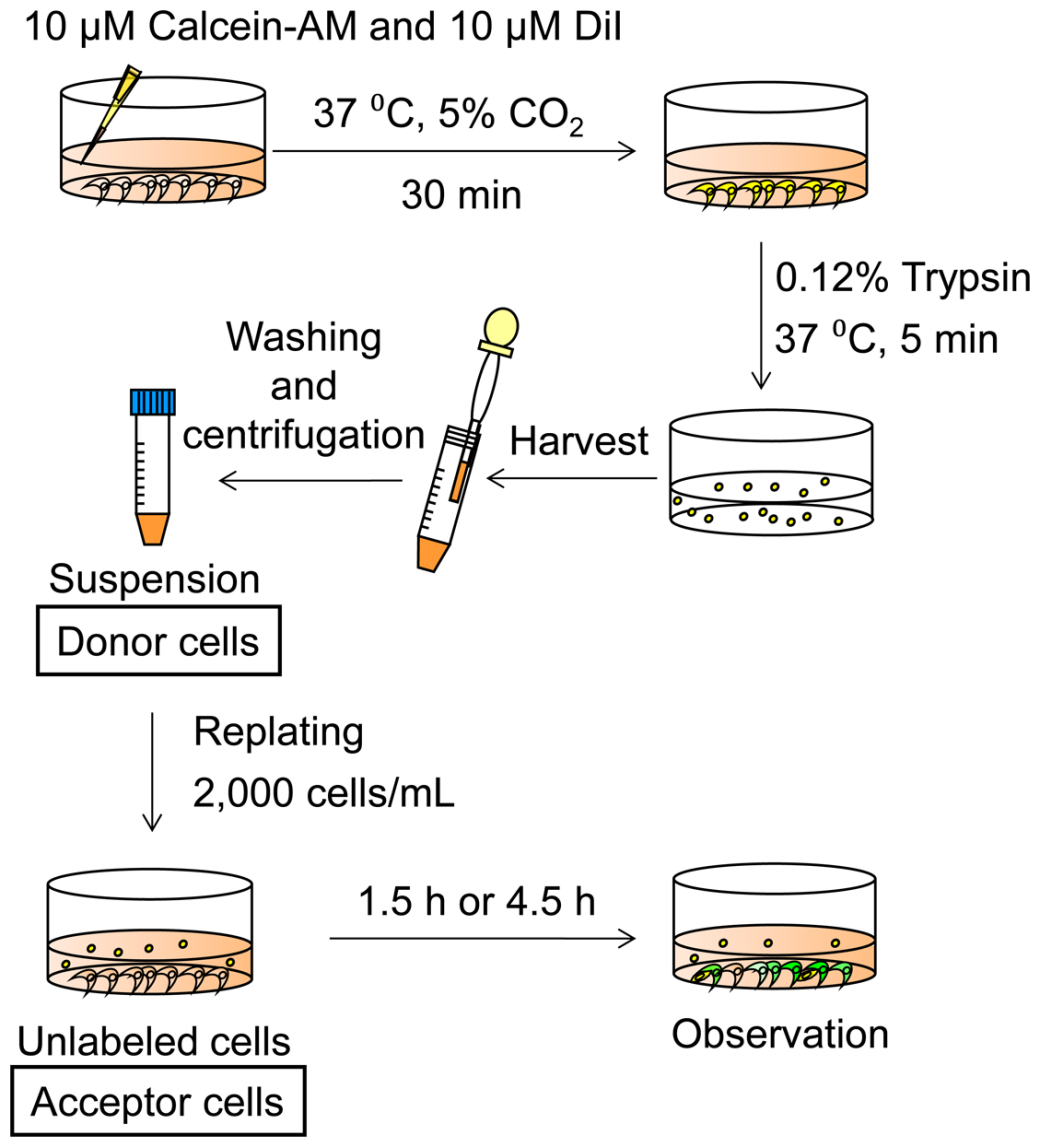
Procedure for fluorescent dye-transfer assay. Cultures of the SLFs prepared from the cochlea of naïve animals were labeled with both DiI (*red*) and calcein-AM (*green*) as donor cells (y*ellow*). The donor cells were then seeded onto unlabeled cells (acceptor cells), and the cultures were incubated for 1.5 or 4.5 h.

Cultures of SLFs were labeled simultaneously with calcein-AM and DiI as donor cells. Calcein-AM is cleaved by cytosolic esterases and then becomes able to permeate GJs. DiI associates with cell membranes and fails to be transferred to unlabeled cells [19]. The labeled cells (donor cells) were harvested with a 0.25% trypsin solution for 5 min, rinsed, and seeded onto unlabeled cells, which had been cultured as acceptor cells. These cells were then cultured for 4.5 h to allow the donor cells to establish GJ-IC with the acceptor cells (Figure 1). The number of cells receiving calcein from the donor cells provides an index of GJ-IC. The transfer of calcein to the acceptor cells from the donor cells was completely blocked by pretreatment for 4 h with 200 µM carbenoxolone, which completely and reversibly blocks the GJ-IC under certain experimental conditions [19].

### Data analysis

The area under the curve was calculated by analyzing the densitometric data by the software Lane Analyzer ver. 3 (Copyright Rise Co., Ltd. & Atto Corp. 1999–2001). Each result was expressed as the mean ± S.E.M., and the statistical significance of differences was determined by one-way ANOVA with the Bonferroni/Dunnett *post hoc* test or the Mann-Whitney *U*-test.

## Results

### Localization of Cx26, Cx30, and Na^+^, K^+^-ATPase α1 in the lateral wall structures

**Figure 2:**
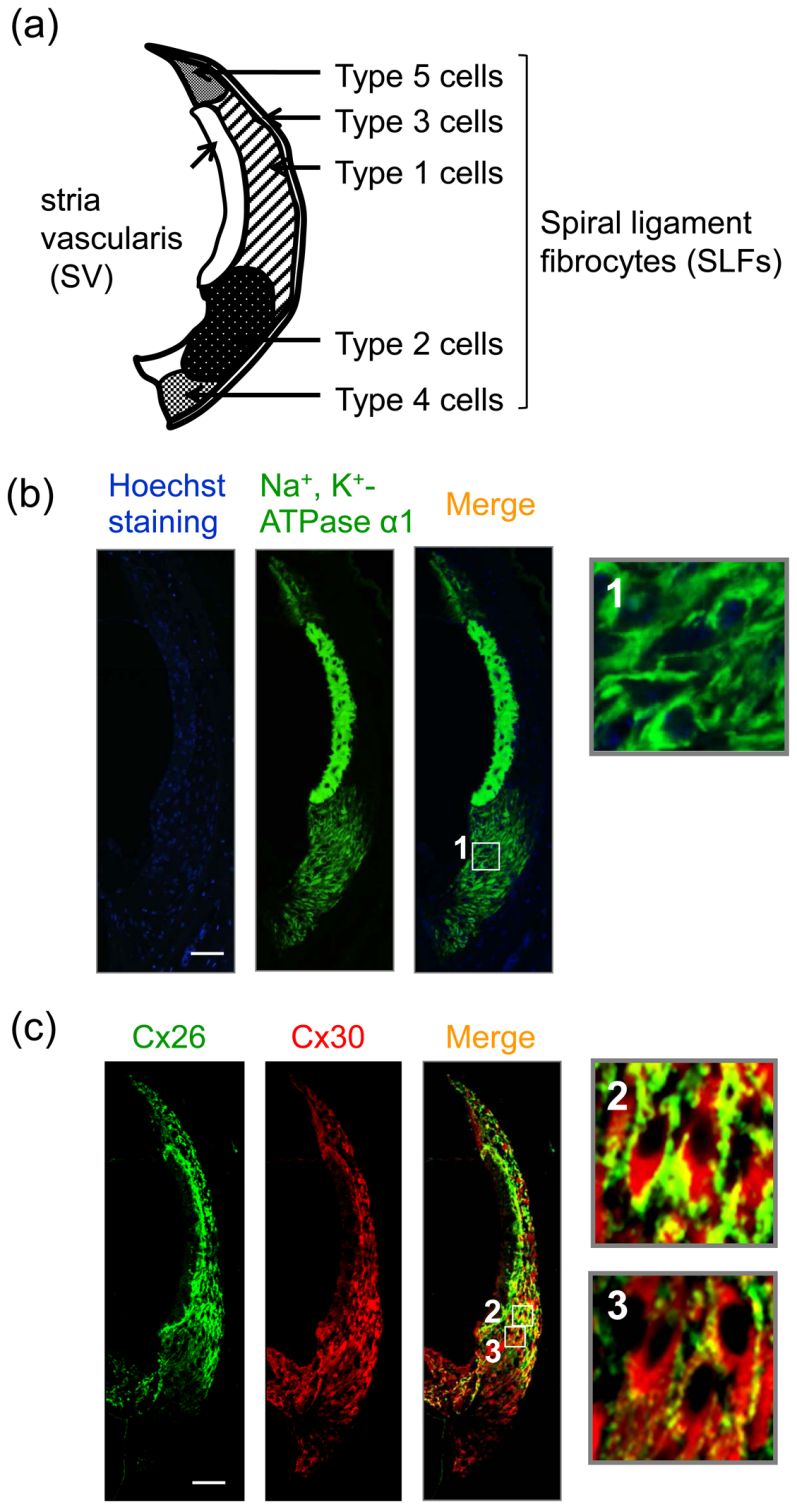
Localization of Cx26, Cx30, and Na^+^, K^+^-ATPase α1 in the lateral wall structures. Mice at the postnatal age of 5 weeks were fixed for preparation of cochlear slices, which were then stained with Hoechst 33342 and immunostained for Cx26, Cx30, and Na^+^, K^+^-ATPase α1 in the lateral wall structures. (**a**) Diagram of various types (types 1–5) of fibrocytes in the lateral wall structures. (**b**) Typical images of Hoechst 33342 staining (*blue*) and Na^+^, K^+^-ATPase α1 immunostaining (*green*) in the lateral wall structures of mice. The rightmost panel is a higher magnification image of the framed squares “1” in the adjacent image. (**c**) Typical images of immunostaining for Cx26 (*green*) and Cx30 (*red*) in the lateral wall structures of mice at the postnatal age of 5 weeks. The rightmost panels are higher magnification images of the framed squares “2” and “3” in the adjacent merged image. Yellow color denotes co-localization of Cx26 and Cx30. These experiments were carried out at least 4 times under the same experimental conditions, with similar results. Scale bar  = 50 µm.

Multiple isoforms of 3 subunits (α, β, and γ) comprise the Na^+^, K^+^-ATPase oligomer. Of these subunits, the α subunit has the binding sites for ATP and cations (Na^+^ and K^+^). The cochlear GJs are assembled with Cx26 and Cx30 [6], [7]. Thus, we examined the expression of ion-transport proteins including Cx26, Cx30, and the α1 subunit of Na^+^, K^+^-ATPase in the lateral wall structures of naïve adult mouse. Histological assessment by immunostaining revealed that Na^+^, K^+^-ATPase α1 was located predominantly in the type 2/4 SLFs and in the entire SV (Figure 2b; see Figure 2a for locations of these cells or structure). Cx26 was expressed mainly in the type 1 cells and in the basal cells of the SV with weak expression in the type 2/4 cells; whereas Cx30 was found in all SLFs and in the basal cells of the SV (Figure 2c). Co-localization of Cx26 and Cx30 was observed in the adhesion sites of the SLFs (Figure 2c at high magnification).

### Developmental changes in the expression of Cx26, S100β, and Na^+^, K^+^-ATPase α1 in the lateral wall structures

**Figure 3:**
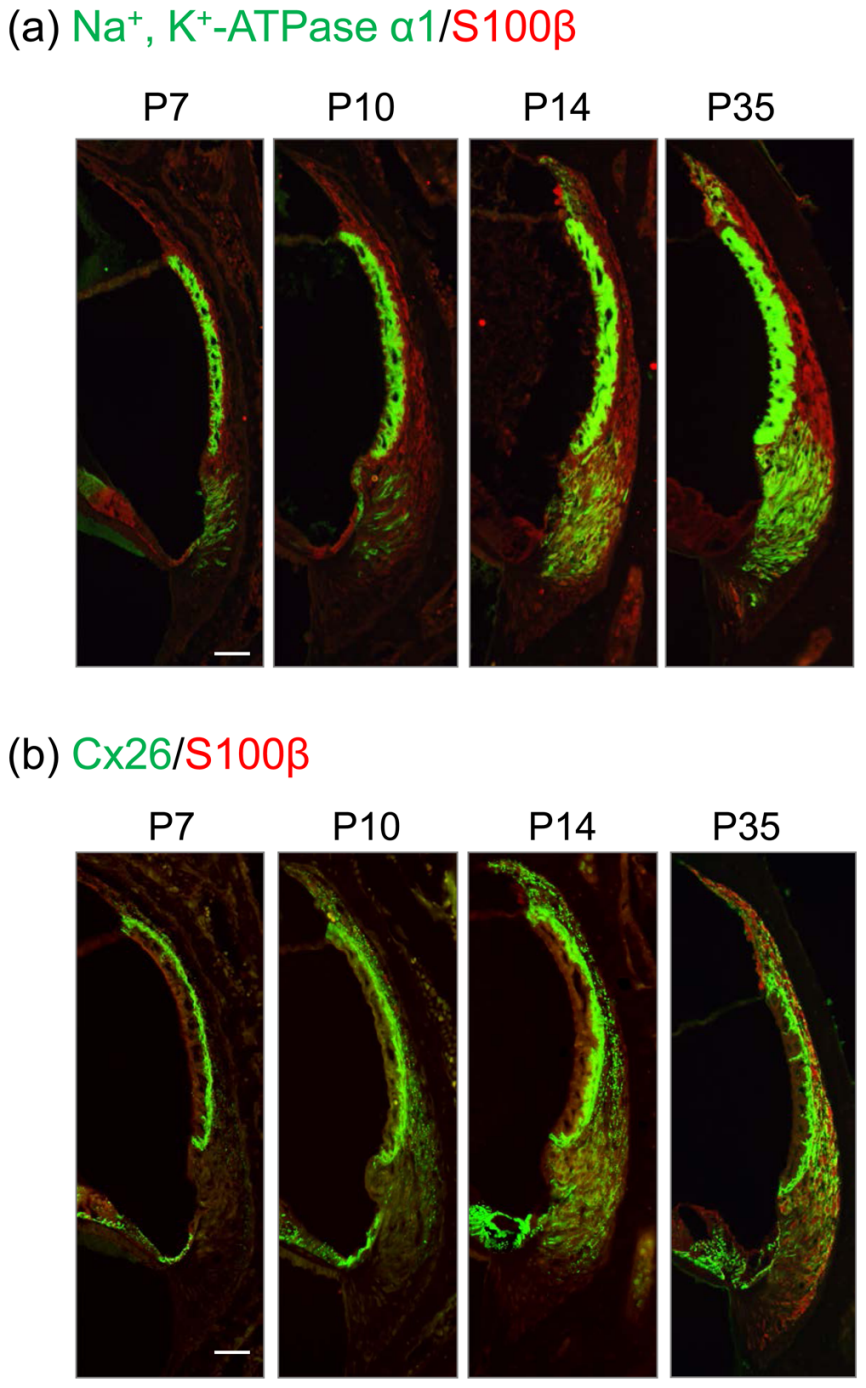
Developmental changes in Cx26, S100β, and Na^+^, K^+^-ATPase α1 in the lateral wall structures. Mice of various postnatal ages were fixed for preparation of cochlear slices, which were then stained with Hoechst 33342 and immunostained for Cx26, Na^+^, K^+^-ATPase α1, and S100β in the lateral wall structures. (**a**) Typical images of immunostaining for Na^+^, K^+^-ATPase α1 (*green*) and S100β (*red*) in the lateral wall structures of mice at P7 to P35. (**b**) Typical images of immunostaining for Cx26 (*green*) and S100β (*red*) in the lateral wall structures of mice at P7 to P35. These experiments were carried out at least 4 times under the same experimental conditions, with similar results. Scale bar  = 50 µm.

A previous report demonstrated that the various types of SLFs in the postnatal rat have different processes of proliferation and differentiation [20]. Using immunostaining, thus, we determined the expression levels of Na^+^, K^+^-ATPase α1 and Cx26 in the lateral wall structures at the postnatal ages of day 7 (P7), P10, P14, and P35 (Figure 3). S100β is a family protein of S100, which is known to express in the type 1/2 cells of the SLFs [21]. Na^+^, K^+^-ATPase α1 appeared in the type 2/4 SLFs and in the SV even at P7. The level of Na^+^, K^+^-ATPase α1 was very weak at P7 and gradually increased up to the adult age (P35, Figure 3a). Cx26 was found mainly in the basal cells of the SV, but not in the SLFs, even at P7. At P10, Cx26 started to appear in the type 1 cells with the level increasing up to P35 (Figure 3b).

### Effect of noise exposure on mRNA level of GJ genes in the SL

**Figure 4:**
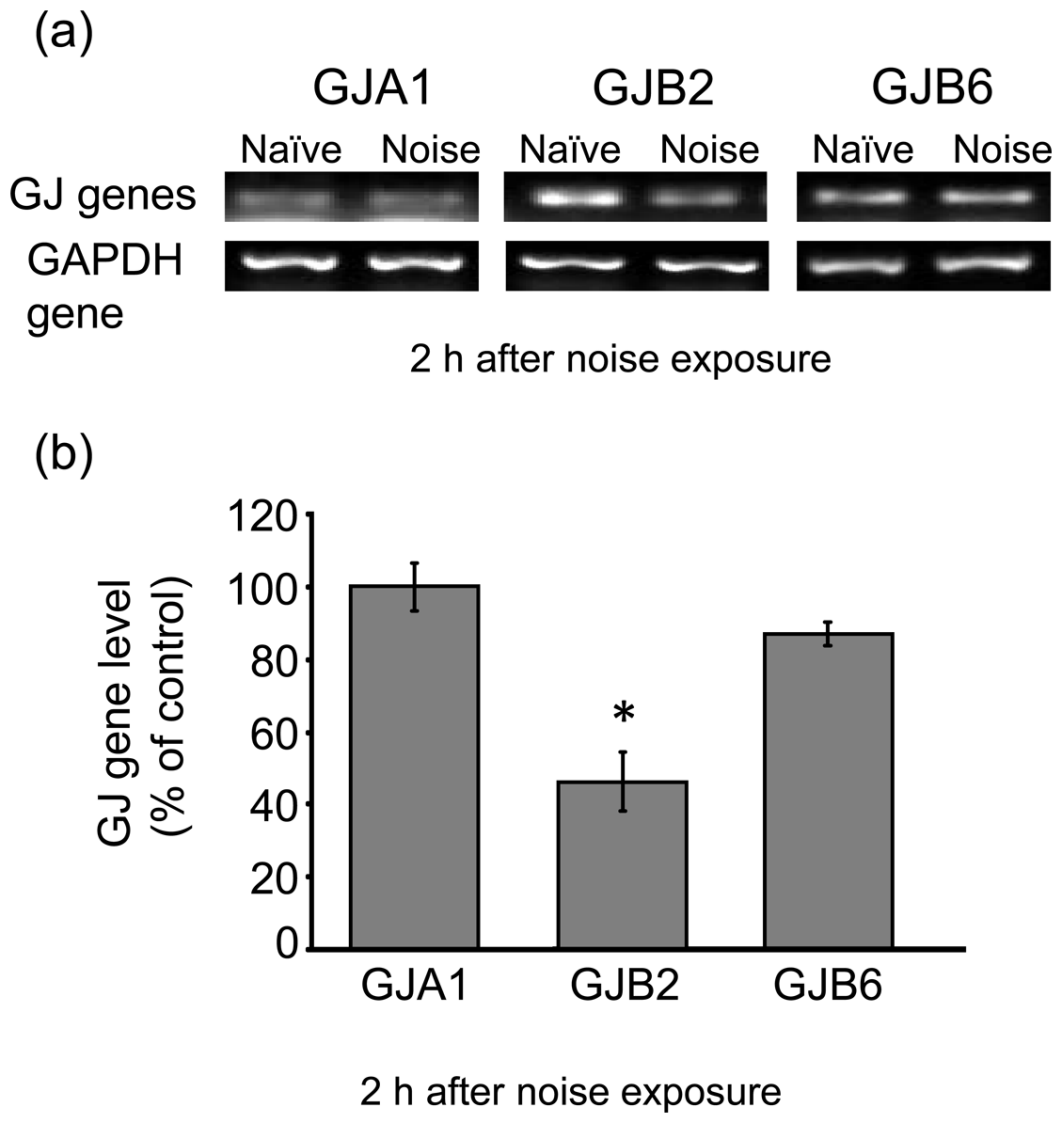
Effect of noise exposure on the mRNA level of Cxs in the SL. Animals were exposed to noise at 110-h post-exposure, total RNA was isolated from the SL and subjected to semi-quantitative RT-PCR (**a**) and real-time PCR (**b**) analyses for determination of the mRNA level of GJA1 (Cx43), GJB2 (Cx26), GJB6 (Cx30), and GAPDH. Values are the mean ± S.E. from 5 separate animals per each group. *P<0.05, significantly different from control value obtained for naïve animals.

To determine hearing loss and hair-cell damage following noise exposure, we measured the ABR immediately and on day 7 after a 1-h exposure to noise at 110 dB SPL. Noise was effective in shifting the ABR threshold at the frequencies of 4, 12, and 20 kHz immediately after exposure. Immediately post-exposure, the ABR threshold shifts were from 40 to 80 dB at all frequencies. The ABR threshold shifts induced by noise remained more than 20 dB even on day 7 post-exposure (data not shown). These results suggest that noise at 110 dB SPL produced a permanent threshold shift. Under these experimental conditions, we examined the effect of noise exposure on the mRNA level of GJA1, GJB2, and GJB6, which are the coding genes for Cx43, Cx26, and Cx30, respectively. Semi-quantitative RT-PCR (Figure 4a) and real-time PCR (Figure 4b) both revealed a decrease in the mRNA level of GJB2 in the SL at 2-h post-noise exposure. However, no significant change was seen in the mRNA levels of GJA1 and GJB6 there under the same experimental conditions.

### Effect of noise exposure on the protein levels of Cx26 and Cx30 in the SL

**Figure 5:**
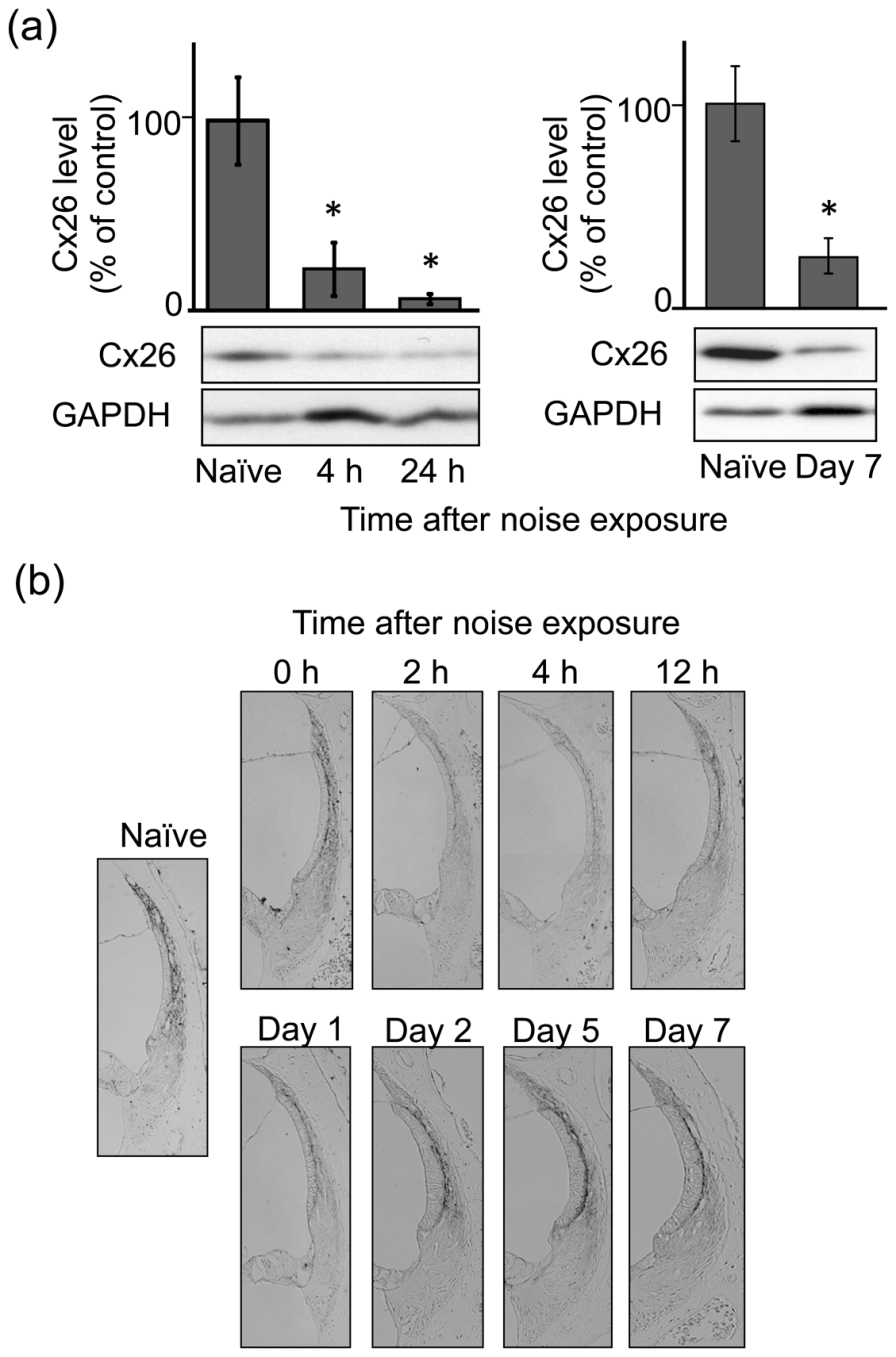
Effect of noise exposure on the protein level of Cx26 in the SL. Animals were exposed to noise at 110(**a**) Tissue lysates were prepared from the SL of the cochlear at the various time points indicated post-noise exposure as well as from that of naïve animals for determination of Cx26 levels by immunoblot analysis. Values are the means ± S.E. from 4–7 separate animals per each group. *P<0.05, significantly different from control value obtained for naïve animals. (**b**) At the various time points indicated post-exposure, animals were fixed for preparation of the cochlear slices, which were then immunostained for Cx26 in the lateral wall structures of the cochlea. These experiments were carried out at least 6 times under the same experimental conditions, with similar results. 0 h, immediately after noise exposure.

We next determined the protein levels of Cx26 and Cx30 in the SL of naïve and noise-exposed animals. Figure 5 shows the Cx26 level at various time points following noise exposure. Immunoblot analysis revealed that noise exposure produced a dramatic decrease in the Cx26 level at 4 h, 24 h, and on day 7 post-exposure (Figure 5a). In addition to immunoblot analysis, immunostaining revealed this noise-induced decrease in the Cx26 level in the type 1 cells of the SL and in basal cells of the SV from at least 2 h to 24 h (day 1) post- exposure (Figure 5b). The decrease in Cx26 in the SV remained even later on day 7, although the Cx26 level in the basal cells returned until the naïve one from days 2 to 7 post-exposure.

**Figure 6:**
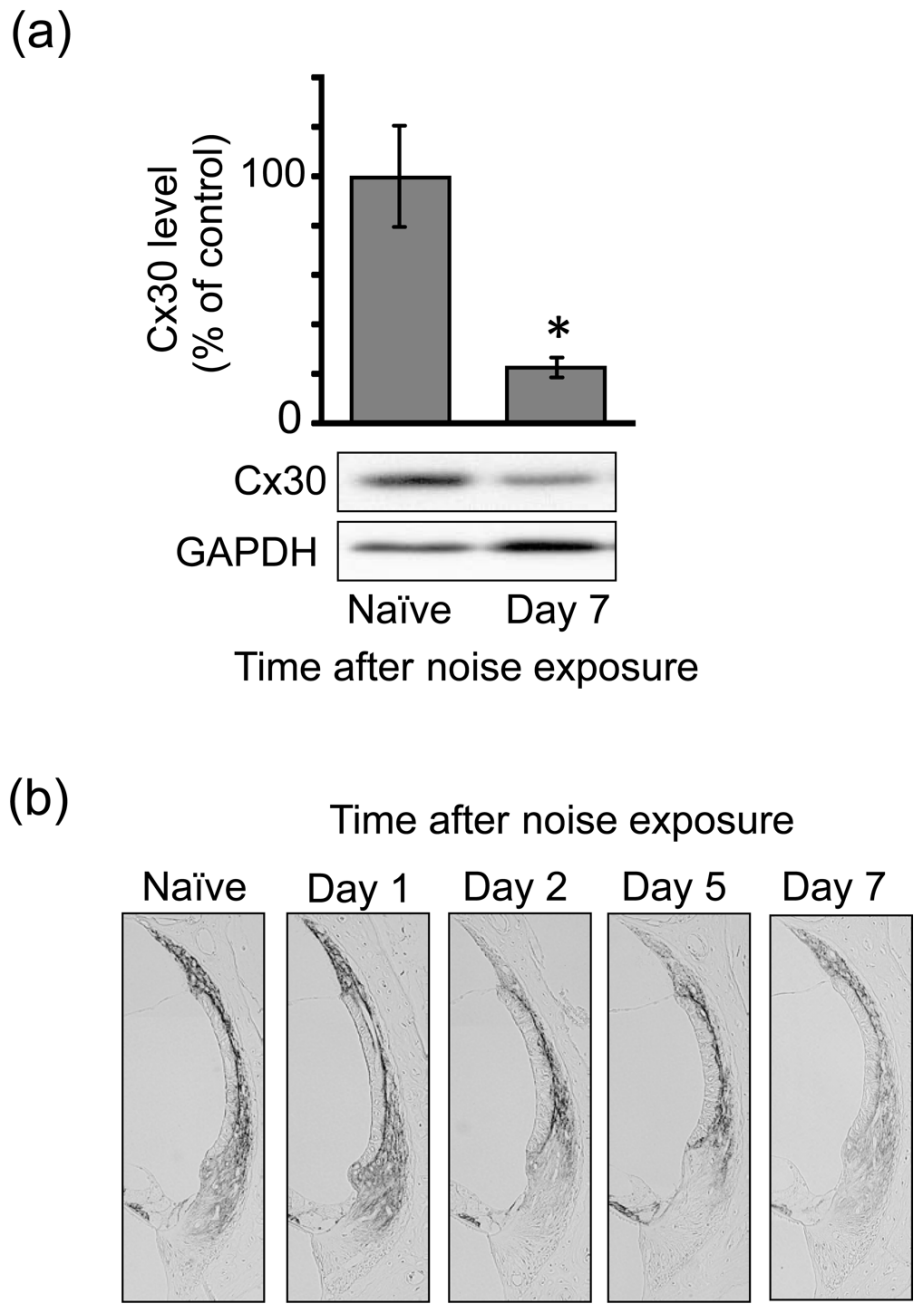
Effect of noise exposure on the protein level of Cx30 in the SL. Animals were exposed to noise at 110(**a**) Tissue lysates were prepared from the SL of the cochlea on day 7 post-noise exposure as well as from that of naïve animals for determination of Cx30 levels by immunoblot analysis. Values are the means ± S.E. from 4 separate animals per each group. *P<0.05, significantly different from control value obtained for naïve animals. (**b**) At the various time points indicated post-exposure, animals were fixed for preparation of cochlear slices, which were then immunostained for Cx30 in the lateral wall structures of the cochlea. These experiments were carried out at least 6 times under the same experimental conditions, with similar results.

Figure 6 shows the Cx30 level at various time points following noise exposure. Immunoblot analysis using the protein samples prepared from the SV revealed that Cx30 expression markedly decreased at least on day 7 post-exposure (Figure 6a). This noise-induced decrease in the level of Cx30 protein was found in the type 2/4 cells on day 2 and afterwards, followed by that in the type 1 cells on day 7 post-exposure (Figure 6b).

### Effect of noise exposure on Na^+^, K^+^-ATPase activity in the SL

**Figure 7:**
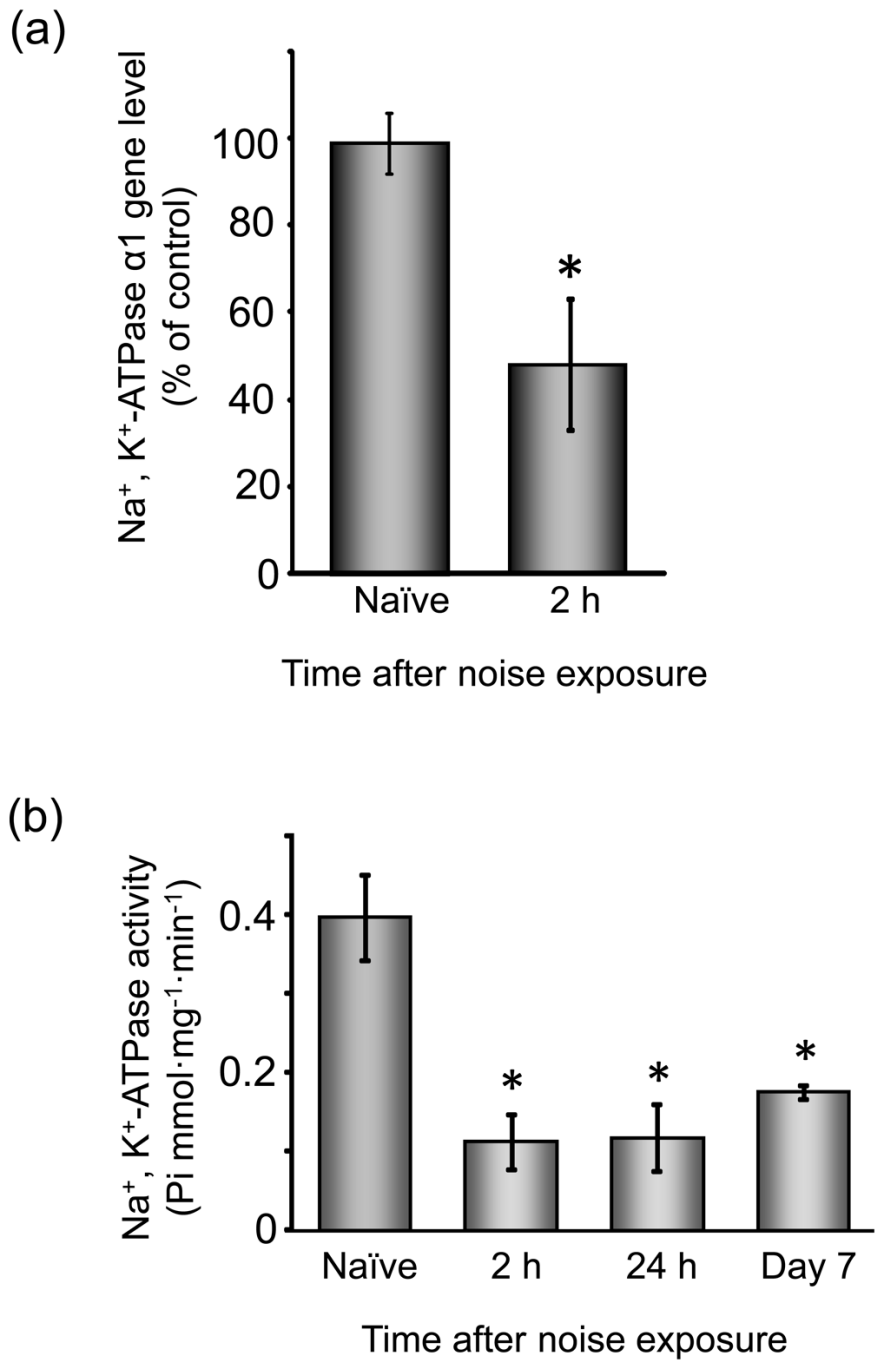
Effect of noise exposure on the mRNA level and activity of Na^+^, K^+^-ATPase in the SL. (**a**) Animals were exposed to noise at 110 dB SPL for 1 h. At 2 h post-exposure, total RNA was isolated from the cochlear SL and subjected to real-time PCR analyses for determination of the mRNA level of Na^+^, K^+^-ATPase α1 and GAPDH. (**b**) Tissue lysates were prepared from the cochlear SL at the various time points indicated post-noise exposure as well as from that of naïve animals for determination of Na^+^, K^+^-ATPase activity. Values are the mean ± S.E. from 5–10 separate animals per each group. *P<0.05, significantly different from control value obtained for naïve animals.

To evaluate whether Na^+^, K^+^-ATPase changed as a noise-induced event in the lateral wall structures, we determined the mRNA level of Na^+^, K^+^-ATPase α1 and Na^+^, K^+^-ATPase activity in tissue lysates prepared from the SL of naïve and noise-exposed animals (Figure 7). Noise exposure dramatically decreased both the mRNA level of Na^+^, K^+^-ATPase α1 and Na^+^, K^+^-ATPase activity at least 2 h post-exposure. Importantly, this noise-induced decrease was maintained at least up to day 7 post-exposure.

### Effect of tempol and L-NAME on noise-induced permanent hearing loss and noise-induced events in the SL

**Figure 8:**
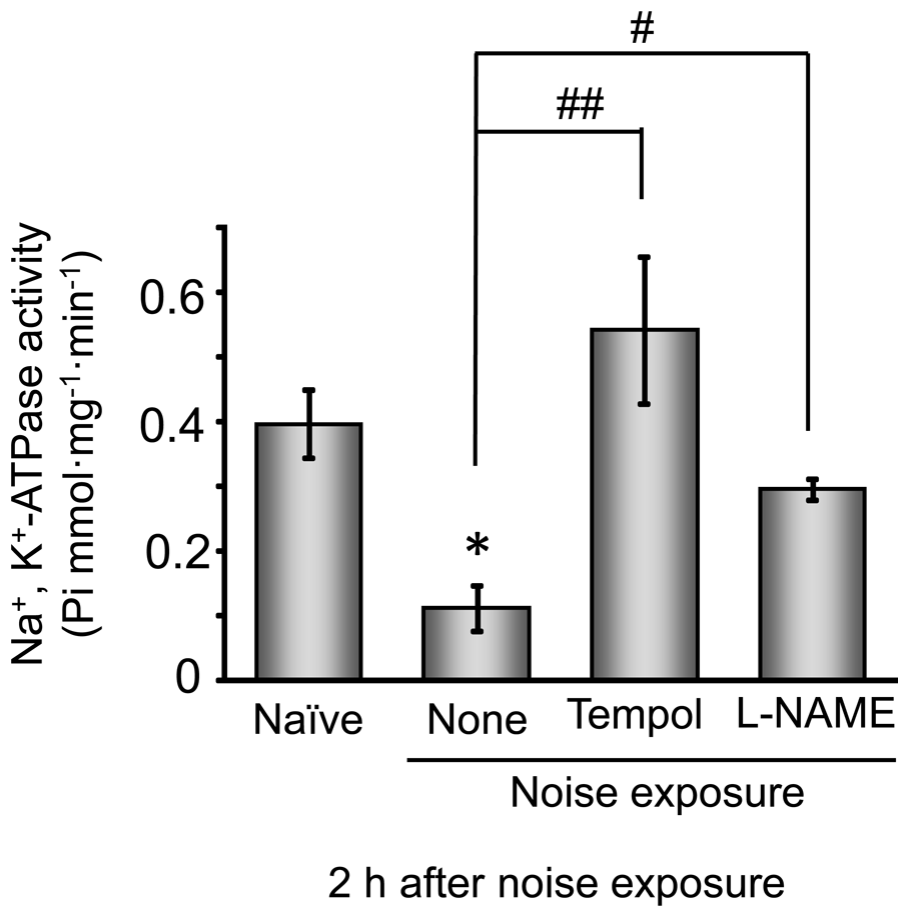
Effect of tempol and L-NAME on Na^+^, K^+^-ATPase activity in the SL. Mice were exposed to noise at 110(30 mg/kg, i.p.) or L-NAME (1 mg/kg, i.p.). Tissue lysates were prepared from the SL for determination of Na^+^, K^+^-ATPase activity. Values are the mean ± S.E. from 3–10 separate animals per each group. *P<0.05, significantly different from control value obtained for naïve animals. ^#^ *P*<0.05, ^##^ *P*<0.01, significantly different from the value obtained for noise-exposed animals without drug treatment.

Evidence for involvement of oxidative stress in noise-induced hearing loss comes from our previous findings that noise-induced hearing loss is prevented by treatment with tempol and L-NAME, which are a reactive oxygen species scavenger and nitric oxide synthase inhibitor, respectively [16]. To evaluate if oxidative stress was involved in the noise-induced decreases in the Cx26 level and Na^+^, K^+^-ATPase activity, we examined the effect of tempol and L-NAME applied separately on the noise-induced decreases in Cx26 level and Na^+^, K^+^-ATPase activity in the SL, as well as on the noise-induced shift in the ABR threshold, under the same experimental conditions. Noise exposure produced a significant decrease in the Cx26 level and a remarkable shift in the ABR threshold (Table 1). As expected, tempol and L-NAME had the ability to abolish both events induced by noise exposure. In addition to Cx26, the noise-induced decrease in Na^+^, K^+^-ATPase activity was prevented by tempol or L-NAME at the same doses as effective in abolishing the threshold shift (Figure 8).

**Table 1:** Effect of treatment with tempol or L-NAME on ABR threshold shift and the decrease in Cx26 level in noise-exposed mice.

Treatment	Cx26 level (% of control)	ABR threshold shift (dB)			
		Time point of measurement	4 kHz	12 kHz	20 kHz
None	21.9±13.9**	Immediately	50.3±5.5	63.8±5.0	63.8±5.2
		Day 7	20.2±1.5	33.0±2.6	33.0±2.6
Tempol	53.8±17.9#	Immediately	41.7±4.4#	41.7±1.7#	43.3±1.7##
		Day 7	1.7±1.7##	5.0±0.1##	10.0±2.9##
L-NAME	45.2±4.2##	Immediately	38.3±1.7#	45.0±2.9##	43.3±1.7##
		Day 7	3.3±.7##	10.0±0.1##	8.3±1.7##

Mice were exposed to noise at 110 dB SPL for 1 h. At 30 min before the onset of noise exposure, the animals were given tempol (30 mg/kg, i.p.) or L-NAME (1 mg/kg, i.p.). The ABR threshold was assessed at the frequencies of 4, 12, and 20 kHz on immediately and day 7 post-noise exposure. Values are the mean ± S.E. from 3–12 separate animals per each group. For determination of the Cx26 level by immunoblot analysis, tissue lysates were prepared from the SL at 4 h post-noise exposure. Values are the mean ± S.E. from 4 separate animals per each group.

**P<0.01, significantly different from control value obtained for naïve animals.

# *P*<0.05,

## *P*<0.01, significantly different from the value obtained for noise-exposed animals without drug treatment.

### Effect of 4-HNE on the GJ-IC and Cx26 level in SLF cultures

**Figure 9:**
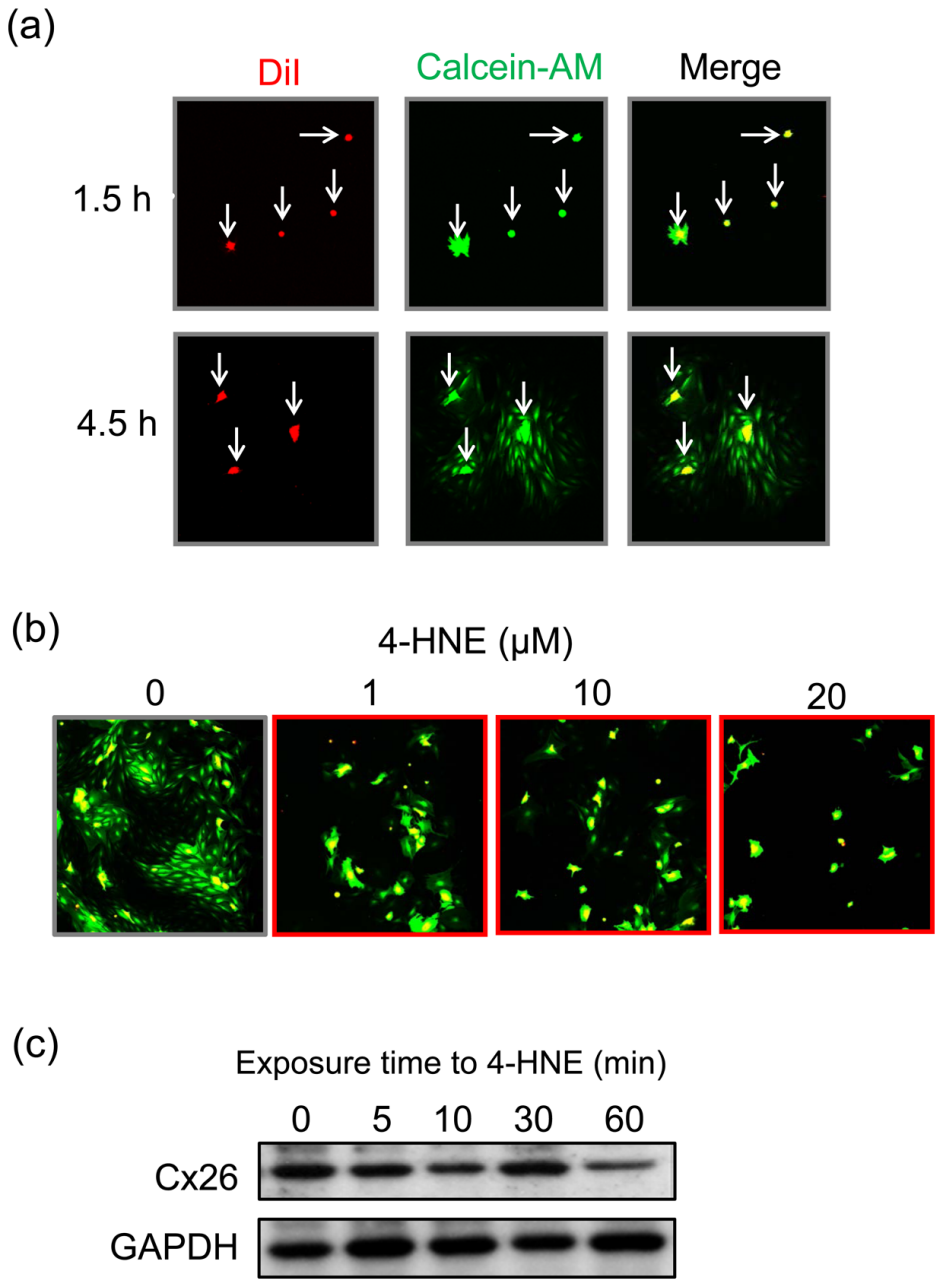
Effect of 4-HNE on GJ-IC and Cx26 level in SLF cultures. Cultures of the SLFs prepared from the cochlea of naïve animals were subjected to fluorescent dye-transfer assay for determination of GJ-IC. The SLFs were labeled with both DiI (*red*) and calcein-AM (*green*) as donor cells. The donor cells were then seeded onto unlabeled cells (acceptor cells), and the cultures were incubated for 1.5 or 4.5 h in the absence or presence of 4-HNE. (**a**) Time-dependent intercellular trafficking of calcein AM in the SLFs without treatment with 4-HNE. White arrows denote the donor cells. (**b**) The cells were incubated for 4.5 h in the presence of either vehicle or 4-HNE at the different concentrations indicated. (**c**) The level of Cx26 in the SLFs was determined by immunoblot analysis at the various time points indicated after the addition of 4-HNE at 10 µM. These experiments were carried out at least 4 times under the same experimental conditions, with similar results.

Our previous report demonstrated that 4-HNE-protein adducts, the 4-HNE of which is the major aldehydic product of lipid peroxidation and believed to be largely responsible for the cytopathological effects observed during oxidative stress [22], are produced in the lateral wall structures following noise exposure under the same experimental conditions as used in the present study [16]. To elucidate the effect of 4-HNE on GJ-IC in the SLFs of the cochlea, we used a fluorescent dye-transfer assay to determine whether an exposure to 4-HNE would affect GJ-IC in cultures of SLFs. Figure 9a shows fluorescence micrographs taken at 1.5- and 4.5-h incubation in the absence of 4-HNE. Both DiI (*red*) and calcein-AM (*green*) were observed in the donor cells, but almost not in the acceptor cells, at 1.5-h incubation. At 4.5-h incubation, expectedly, calcein-AM spread from the donor cells to the adjacent acceptor cells, indicating establishment of GJ-IC in the cultures. Under the same experimental conditions, an exposure to 4-HNE at the concentration of 1 to 20 µM dramatically decreased the spread of calcein-AM to the acceptor cells in a concentration-dependent manner (Figure 9b). The level of Cx26 was gradually decreased by the exposure to 4-HNE almost in an exposure time-dependent manner (Figure 9c). 4-HNE failed to show any signs of cytotoxicity toward the SLFs under the same experimental conditions (data not shown).

### Effect of 4-HNE on Na^+^, K^+^-ATPase in SLF cultures

**Figure 10:**
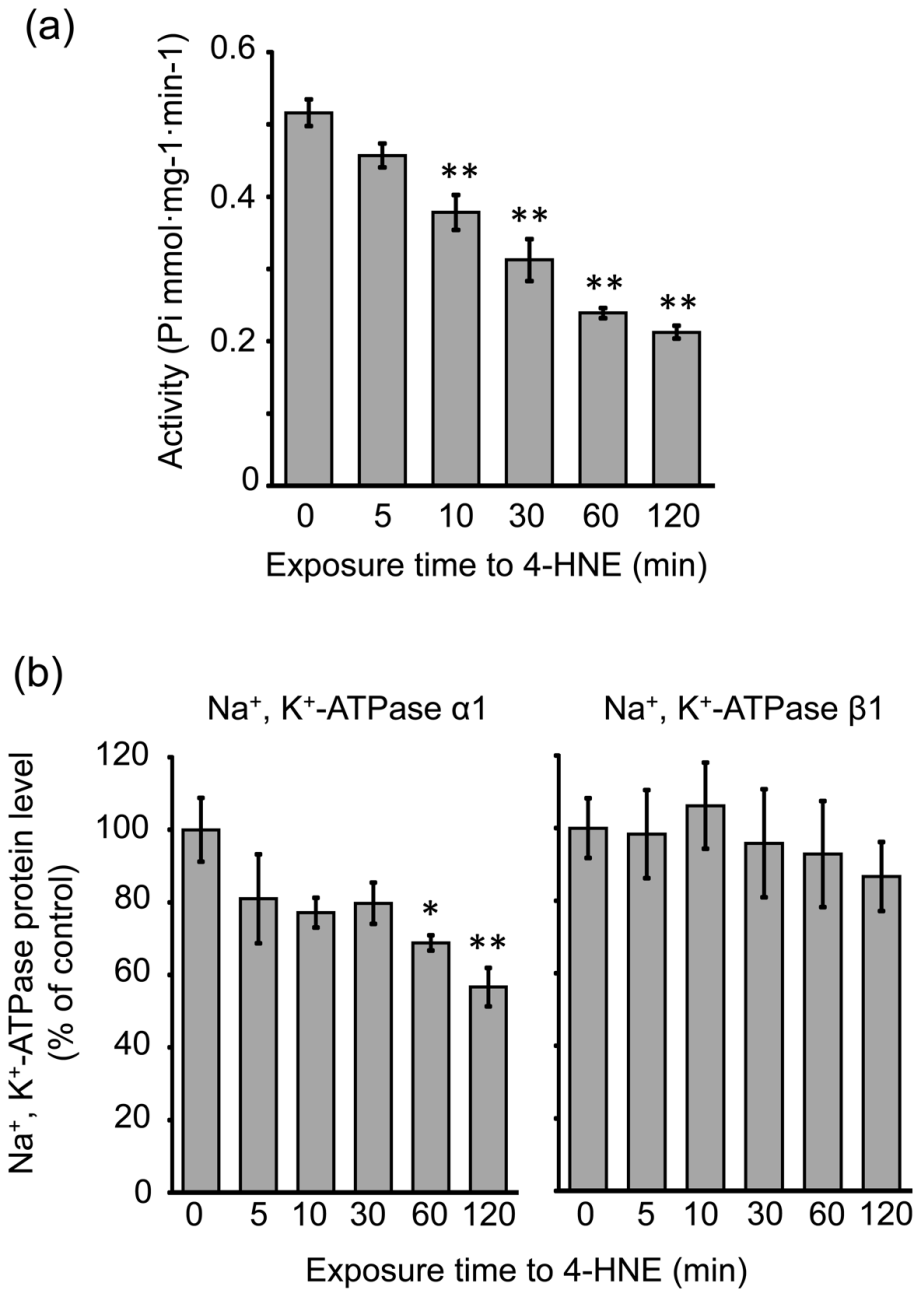
Effect of 4-HNE on Na^+^, K^+^-ATPase in SLF cultures. Cultures of the SLFs prepared from the cochlea of naïve animals were incubated for the indicated times in the presence of 4-HNE at 0.1 µM and then examined for Na^+^, K^+^-ATPase activity (**a**) and the protein level of Na^+^, K^+^-ATPase α1 and β1 subunits (**b**). Values are the mean ± S.E. from 4 independent experiments. *P<0.05, significantly different from control value obtained for untreated cells.

To elucidate the involvement of 4-HNE in the noise-induced decrease in Na^+^, K^+^-ATPase activity in the SL, we assessed the change in Na^+^, K^+^-ATPase activity and protein levels of Na^+^, K^+^-ATPase subunits in the cultured SLFs during the culture in the absence or presence of 4-HNE. Exposure to 4-HNE at the concentration of 1 µM significantly decreased Na^+^, K^+^-ATPase activity by a 10-min exposure or longer (Figure 10a). Under the same experimental conditions, the protein level of the Na^+^, K^+^-ATPase α1 subunit was significantly decreased by exposure to 4-HNE in a exposure time-dependent manner (Figure 10b). However, no significant change by exposure to 4-HNE, at least when used at 1 µM, was seen in the protein level of the Na^+^, K^+^-ATPase β1 subunit.

## Discussion

The primary aim of the present study was to determine whether disruption of the ion- trafficking system such as GJ-IC and Na^+^, K^+^-ATPase activity in the lateral wall structures of the cochlea would be involved in the process producing hearing loss. To this end, we used an *in vivo* model of hearing loss induced by the exposure to intense noise, as well as the primary cultures of SLFs as an *in vitro* system. In the *in vivo* model, previously established by us, noise at different SPLs produced hearing loss with a temporary or permanent threshold shift of the ABR in an SPL-dependent manner [16]. Noise at lower SPL (90 and 100 dB) or higher SPL (110 and 120 dB) produced hearing loss with a temporary or permanent threshold shift, respectively. Indeed, our current data showing that noise at 110 dB SPL produced permanent hearing loss strongly supports our previous findings. In the present study, we demonstrated at the first time that a prolonged decrease in the levels of GJ-comprising proteins, Cx26 and Cx30, and Na^+^, K^+^-ATPase activity occurred in the lateral wall structures prior to hearing loss induced by acoustic overstimulation. These events prior to the hearing loss were abolished by tempol, a free radical-scavenging agent, which ameliorated the hearing loss under the same experimental conditions. These results allow us to propose that disruption of ion-trafficking including GJ-IC and active transport of Na^+^ and K^+^ in the lateral wall structures contributed to the permanent hearing loss induced by acoustic overstimulation. In addition to the findings from the *in vivo* model, those from the *in vitro* system using SLFs in primary culture demonstrated at the first time that one of the main mediators of oxidative stress, 4-HNE, had the ability to cause dysfunction of GJ-IC and attenuation of Na^+^, K^+^-ATPase activity effected by down-regulation of the Na^+^, K^+^-ATPase α1 level. Based on our previous findings that 4-HNE is produced in the lateral wall structures prior to hearing loss induced by intense noise [16], it is possible that 4-HNE produced by noise expose contributed to hearing loss through disruption of the ion-trafficking system in the lateral wall structures of the cochlea. However, other oxidants may be involved in noise-induced disruption of the ion-trafficking system in the lateral wall structures of the cochlea. Further studies may elucidate any oxidants involved in these events induced by acoustic overstimulation.

### Ion-trafficking system in the lateral wall structures and hearing ability

Endolymph contains 150 mM K^+^, 2 mM Na^+^, and 20 µM Ca^2+^ and maintains the EP at +80 mV, which is crucial for maintenance of hearing ability [23]. The EP enhances the sensitivity of hair cells by increasing the driving force for K^+^ influx and Ca^2+^ permeation, which amplify the motility of hair bundles [24], [25]. It is proposed that noise exposure-induced hearing loss is at least in part due to a reduction in the EP [12], [26], [27]. After K^+^ enters into the hair cells in the organ of Corti by acoustic stimulation, K^+^ must cycle back to the endolymph through the pathway comprising perilymph, supporting cells, and lateral wall structures [1], [28], [29]. In the lateral wall structures, the ion-trafficking system comprising Na^+^, K^+^-ATPase, ion transporters, and GJs contributes to the K^+^ transport and is essential for generation of the EP. The current data showed that Na^+^, K^+^-ATPase and Cx26 reached high levels in the lateral wall structures at P14 (Figure 3a and 3b). Considering that the onset of hearing ability in rodents occurs at postnatal days 13–14 (P13–14), our current data support the proposition that Na^+^, K^+^-ATPase and GJs play an important role for establishing and maintaining the EP and hearing ability. Thus, our present proposition that the decreases in Cx contents and Na^+^, K^+^-ATPase activity in the lateral wall structures, indicating disruption of the ion-trafficking system, induced hearing loss through would be feasible.

### Cxs and noise-induced hearing loss

Non-sensory cells in the cochlea are connected extensively by GJs that facilitate intercellular ionic and biochemical coupling. Several different Cx proteins have been reported to be expressed in the mammalian inner ear. These include Cx26, Cx30, Cx31, and Cx43 [6], [30], [31], [32]. Cx26 and Cx30 are the prominent members co-assembled in most of the cochlear GJs [6]. In the lateral wall structures of mice at adult age (P35), co-expression of Cx26 and Cx30 was observed in the type 1 SLFs and basal cells of the SV (Figure 2c). However, the type 2 SLFs had Cx30 but little Cx26. Therefore, the type 1 and type 2 SLFs expressed hetero-GJs composed of Cx26/Cx30 and homo-GJs of Cx30, respectively. Mutations in the genes encoding Cx26 and Cx30 are also known to cause a substantial portion of the cases of human non-syndromic hereditary deafness, which is one of the most common human birth defects [33]. The current data indicated that exposure to noise produced an immediate and prolonged decrease in the level of Cx26 in the type 1 SLFs and basal cell of the SV. In addition, the results of the RT-PCR analysis allows us to propose that such exposure decreased the gene expression of Cx26, but not that of Cx43 or Cx30, at least immediately post exposure. The noise-induced decrease in Cx26 was prevented by the free radical-scavenging agent and the nitric oxide synthase inhibitor, which use improved the noise-induced hearing loss. These findings suggest that the immediate and prolonged decrease in Cx26 was crucial for noise-induced hearing loss through dysfunction of GJ-IC in the lateral wall structures.

To elucidate the involvement of oxidative stress in GJ-IC in the SL, we used primary cultures of the SLFs derived from adult mice. Our previous data indicated that exposure to noise causes marked expression of 4-HNE-adduct proteins in the SL [16]. 4-HNE is known to form Michael adducts with focal adhesion kinase, β-catenin, paxillin, VE-cadherin, ZO-1, and the actin cytoskeleton in endothelial cells [34], indicating that these junction proteins and cytoskeleton are involved in 4-HNE-induced alterations of cell-cell adhesion in endothelial cells. It is also known that 4-HNE directly binds to insulin receptor substrate-1/-2 proteins and degrades it in adipocytes [35]. In addition, our preliminary data using the cultures of the SLFs showed that 4-HNE was co-localized with Cx43, the level of which decreased after 4-HNE exposure (data not shown). In the present study, we demonstrated that 4-HNE has the ability to decrease Cx26 level and to disrupt the GJ-IC, but not to damage, in the SLFs. These results suggest that 4-HNE directly contributes to the disruption of the GJ-IC through the decrease in Cxs in the SLFs. Furthermore, these *in vitro* data allow us to propose the idea that exposure to noise *in vivo* disrupted the GJ-IC in cells of the lateral wall structures by causing a decrease in contents of Cxs induced at least in part by the formation of 4-HNE. However, the mechanism underlying this 4-HNE-induced decrease remains to be explored in the future.

### Na^+^, K^+^-ATPase and noise-induced hearing loss

Multiple isoforms of 3 subunits, α, β, and γ, comprise the Na^+^, K^+^-ATPase oligomer. The α subunit contains the binding sites for ATP and the cations, whereas the glycosylated β subunit ensures correct folding and membrane insertion of the α subunits. The small γ subunit co-localizes with the α subunit in nephron segments, where it increases the affinity of Na^+^, K^+^-ATPase for ATP. The β subunit, but not the γ subunit, is essential for normal activity of Na^+^, K^+^-ATPase. The α subunit is subject to being phosphorylated at select serine residues. Na^+^, K^+^-ATPase also is well known to participate in the active ion transport in the inner ear and to play an important role in maintaining cochlear function [10]–[12]. In the present study, we demonstrated that exposure to noise *in vivo* decreased the activity of Na^+^, K^+^-ATPase in the SL. The current data that both the free radical-scavenging agent and nitric oxide synthase inhibitor abolished the noise-induced decrease in Na^+^, K^+^-ATPase activity allows us to propose that this noise-induced event was due to excess oxidative stress and/or nitric oxide generation. Indeed, the *in vitro* exposure of the SLF cultures to 4-HNE decreased the activity and α1 subunit protein level of Na^+^, K^+^-ATPase. Thus, it seems likely that this 4-HNE-induced decrease in Na^+^, K^+^-ATPase activity was due to the decrease in the α1 subunit level. However, we currently have no idea regarding the mechanism underlying the 4-HNE-induced decrease in the α1 subunit level, although 4-HNE is known to bind to Na^+^, K^+^-ATPase in striatal synaptosomes [36]

## Conclusions

In the present study, we demonstrated at first time that noise-induced hearing loss was at least in part due to the disruption of the ion-trafficking system, involving a decrease in the levels of Cxs and in Na^+^, K^+^-ATPase activity in the lateral wall structures. Formation of 4-HNE by excessive oxidative stress was considered to be operative in the disruption of GJ-IC in the SL. However, it would be required for future studies to validate the hypothesis that 4-HNE is involve in noise-induced hearing loss. In addition, further evaluation of the mechanism underlying the disruption of GJ-IC should contribute to the development of therapeutics for noise-induced hearing loss.
